# Momba: JANI Meets Python

**DOI:** 10.1007/978-3-030-72013-1_23

**Published:** 2021-02-26

**Authors:** Maximilian A. Köhl, Michaela Klauck, Holger Hermanns

**Affiliations:** 8 Eindhoven University of Technology, Eindhoven, The Netherlands; 9 Aalborg University, Aalborg East, Denmark; 10 Saarland University, Saarland Informatics Campus, Saarbrücken, Germany; 11 Institute of Intelligent Software, Guangzhou, China

## Abstract

JANI-model [6] is a model interchange format for networks of interacting automata. It is well-entrenched in the quantitative model checking community and allows modeling a variety of systems involving concurrency, probabilistic and real-time aspects, as well as continuous dynamics. Python is a general purpose programming language preferred by many for its ease of use and vast ecosystem. In this paper, we present *Momba*, a flexible Python framework for dealing with formal models centered around the JANI-model format and formalism. Momba strives to deliver an integrated and intuitive experience for experimenting with formal models making them accessible to a broader audience. To this end, it provides a pythonic interface for model construction, validation, and analysis. Here, we demonstrate these capabilities.

## 1 Introduction

Dealing with formal models encompasses a variety of tasks which can be challenging from time to time—especially for newcomers. Everything starts with the *construction* of a model or a family thereof. Often a textual or other, more formal, description of the scenario to be modeled is already existing, such as a rough sketch of the desired behavior or a circuit diagram. Then, after a formal model has finally been conceived, one has to *validate* that the model actually adequately models what should be modeled. In this regard models are just like any other human artifact, inadequate initially but over time it gets better. Only after confidence in the model has been established, one is able to harvest the benefits by handing over the model to *analysis* tools, e. g., a model checker.

In this paper, we present *Momba*, a flexible Python framework for dealing with formal models. Momba strives to deliver an integrated and intuitive experience to aid the process of model construction, validation, and analysis. It provides convenience functions for the constructions of models effectively turning Python into a syntax-aware macro language enabling the construction of models in a modular fashion. Momba's built-in simulation engine allows gaining

* This work was partially supported by the ERC Advanced Investigators Grant 695614 (POWVER), by the German Research Foundation (DFG) under grant No. 389792660, as part of TRR 248, see https://perspicuous-computing.science, and by the Key-Area Research and Development Program Grant 2018B010107004 of Guangdong Province.

confidence in a model, for instance, by rapidly prototyping a tool for interactive model exploration and visualization, or by connecting it to a testing framework. Finally, thanks to the JANI-model [6] interchange format, several state-of-the-art model checkers and other tools are readily available for analysis. The latest version of Momba is always available on GitHub [1] and the evaluated artifact of this tool demo paper can be found on Zenodo [27].

*Why Momba?* The idea to harvest a general purpose programming environment for formal modelling is not new at all. For instance, the SVL language combines the power of process algebraic modelling with the power of the bourne shell. As part of many CADP installations [12,13], it is in daily use since its inception [11]. Many formal modeling tools also already provide Python bindings [23,10]. Momba tries not to be yet another incarnation of these ideas.

While the construction of formal models clearly is an integral part of Momba, Momba is more than just a framework for constructing models with the help of Python. Most importantly, it also provides features to work with these models such as a simulator or an interface to different model checking tools. At the same time, it is not just a binding to an API developed for another language, say C++. Momba is *tool-agnostic* and aims to provide a pythonic interface for dealing with formal models while leveraging existing tools. Momba covers the whole process from model creation through validation to analysis. To this end, it is centered around the well-entrenched JANI-model interchange format.

*Why JANI?* Traditionally, most analysis tools for formal models came with their own modeling languages and formats. The resulting fragmentation hindered interoperability between and comparability across different tools. JANI-model [6] has been conceived with the vision to put an end to this fragmentation. It has since been adopted by many quantitative model checkers [20,21,9] while for others translators have been developed [20,9] enabling cross-tool comparability and fostering competition within the community [22,19,7]. Recently, JANI has also been discovered by the planning community [24,25].

Momba supports all features of the JANI-model specification and some of its optional extensions. JANI is the natural foundation for a project like Momba. It provides a solid, well-established, and powerful modeling formalism for a variety of different kinds of systems involving concurrency, probabilistic and real-time aspects, as well as continuous dynamics. A JANI model is a network of interacting automata with variables. Attached to a model one can also specify various kinds of probabilistic and timed properties which can then be checked by several model checkers, e. g., ePMC [20], The Modest Toolset [21], and Storm [23]. The broad tool support for JANI models enables us to build upon existing research and to outsource computation-intensive tasks via unified interfaces.

*Why Python?* Python is a popular high-level programming language, preferred by many for its ease of use and ecosystem. Especially within the data-science community, Python is the go-to language for data analysis and machine learning leaveraging tools such as TensorFlow [2] and scikit-learn [29]. Around these tools, scientific general purpose tools such as Jupyter [26] have emerged. Jupyter provides a platform for documenting scientific experiments and their results in a reproducible way combining code, data, and documentation.

Our vision is to harvest Python's ecosystem and the tools developed by the scientific community for dealing with formal models. Imagine, a Jupyter notebook documenting a model, including the code to construct it, with interactive visualizations of the model itself and various analysis results.

By basing our efforts on a popular language that is appreciated by scientists and established in the scientific community, we hope to lower the entry barrier, especially for those outside the formal methods community.

*The User Perspective.* In what follows, we demonstrate multiple facets of Momba using a variant of Racetrack, a well-known benchmark in autonomous AI decision making [4,31] which has recently found its use in several model checking contexts [16,3,15]. too. We go through the entire process from the construction of a family of models through their validation to their analysis. For each step, we highlight what Momba has to offer in terms of effectively supporting the process.

Originally Racetrack has been a pen-and-paper game [14]. A *track* is a two-dimensional grid comprising *start*, *goal*, *wall*, and *blank* cells (cf. Fig. 1) [4]. A vehicle starts off with some initial velocity from a start cell, with the objective to reach a goal cell as fast as possible without crashing into a wall. The vehicle is controlled by nine possible actions modifying the current velocity vector. Racetrack naturally lends itself as a benchmark for sequential decision making in risky scenarios, in particular, when extended with probabilistic noise. In a variety of such noisy forms, it has been adopted as a benchmark for *Markov Decision Process* (MDP) algorithms in the AI community [4,5,28,30,31].

For our demonstration, we consider multiple *variants* of Racetrack giving rise to a family of MDPs, studied recently [3] from a feature-oriented perspective [8]. For example, there are different tank options and fuel is consumed according to various consumption models. In addition, there are different undergrounds inducing probabilistic noise modeling slippery road conditions. Clearly, this modeling scenario is beyond what is possible with mere model parametrization, especially so because we are interested in the car's performance on different tracks each inducing its own MDP [4].

## 2 Scenario-Based Model Construction

Usually, formal models are not constructed out of thin air but based on some kind of scenario description existing upfront. Such descriptions usually comprise an operational characterization of the behavior to model together with additional and sometimes more formal information about the specific case. Our use case is no exemption, here a textual description of the behavior of the car is provided together with a specific track and a specification of the variant.

Naturally, Python can be used to nicely capture the formal parts of a scenario description in various data structures. Combined with a domain-specific parser for configuration files, scenario descriptions are interchangeable and easy to interface with the code for model construction. In our case, a textual representation of the track (cf. Fig. 1) [4] is provided and parsed together with additional

```
dim: 12 35
xxxxxxxxxxxxxxxxxxxxxxxxxxxxxxxxggg
xxxxxxxxxxxxxxxxxxxxxxxxxxxxxxxx...
xxxxxxxxxxxxxxxxxxxxxxxxxxxxxxxx...
xxxxxxxxxxxxxxxxxxxxxxxxxxxxxxxx...
xxxxxxxxxxxxxxxxxxxxxxxxxxxxxxxx...
s..................................
s..................................
s..................................
s..................................
xxxx...............................
xxxxxxxx...........................
xxxxxxxxxxxx.......................
```

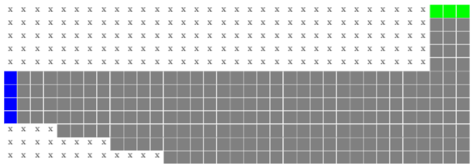

**Fig. 1.** Textual representation (left) and picture of a track (right): start cells in blue (s), goal cells in green (g), and wall cells marked with x.

parameters, like the size of the tank and the type of the underground, into a data structure tailored to that purpose.

Now, how does Momba support the construction of models from such data structures? A distinguishing feature of Momba is that it effectively turns Python into a syntax-aware macro language enabling the modular construction of models. For our Racetrack use case different fuel consumption models can be captured as macros from JANI expressions to JANI expressions:

```
linear = lambda dx, dy: expr("abs($dx) + abs($dy)", dx=dx, dy=dy)
quadratic = lambda dx, dy: expr("$linear ** 2", linear=linear(dx, dy))
```

A macro is simply a Python function. Upon execution, these macros construct JANI expressions using a straightforward syntax inspired by Python expressions. In this case, both functions take expressions for the current velocity of the vehicle in $x$ and $y$ dimension and return an expression for the resulting fuel consumption which is either *linear* or *quadratic* in the velocity. In contrast to how macros work in languages like C, syntax-aware macros using Momba's `expr` function prevent surprises from mere text-based expansion. Being Python functions, macros can be easily passed around and used elsewhere:

```
assignments = {
    "fuel": expr(
        "min(TANK_SIZE, max(0, fuel - floor($consumption)))",
        consumption=fuel_model(car_dx, car_dy),
    )
}
```

Here, we update the fuel level by taking whatever macro has been provided for computing the fuel consumption. This code is part of constructing an edge for the tank automaton in a modular fashion in the sense that the consumption model is exchangeable. Momba provides further functions, for instance, for declaring variables, like `fuel`, and constructing automata, networks, as well as other model objects. Most of these functions provide all kinds of comforts, for instance, directly checking the types of the involved expressions.

Using syntax-aware macros and Momba's other convenience functions, we arrive at a Python script `racetrack.py` [27] generating a collection of JANI models from scenario descriptions comprising a track and specifying a variant. Iterating over possible scenario descriptions, hundreds of JANI models can be generated fully automatically and consequently be analyzed.

## 3 Validation by Simulation

Having our models ready, we have to somehow gain confidence that they actually model what we want them to, before handing them over to analysis tools. One way of gaining confidence into a model is by simulating its behavior and manually checking it for consistency with the own understanding of what the model should do. Just like any kind of debugging, this can be a tedious and frustrating process, especially with text-based traces generated by some generic simulator. Momba instead comprises a built-in simulation engine, enabling rapid development of interactive visualizations. This effectively allows us to steer a vehicle through a track thereby exploring a model's behavior, testing edge cases as in a racing game, and ultimately gaining confidence in the model.

Momba's built-in simulation engine supports the simulation of a variety of different JANI models including timed models. It has been written completely from scratch with easy accessibility from Python in mind. Non-determinism can be resolved by uniform random sampling or by querying an external oracle such as, in the case of our interactive visualization, the user, a testing framework, or even a neural network as done for DSMC [16]. For each step, the simulator provides all the necessary information like the binding of variables to values, the locations the various automata of a network are in, and the possible actions (and time delays for timed models) that can be taken. This information can then be extracted and used to display whatever is of interest for understanding and investigating the behavior of the model under scrutiny.

Fig. 2 shows a simple interactive visualization of the Racetrack example based on Momba's simulation engine where the user can steer the vehicle (indicated by the yellow asterisk) through the track by entering acceleration values. Certainly, there is ample room for beautification of this simulator (see TraceVis [15] for example) but for rapid model development this is not needed. After playing around with the interactive simulation for a while and testing various edge cases, we are confident that the model is adequate.

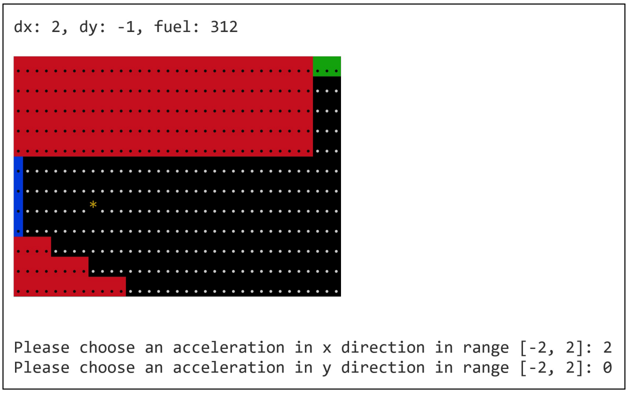

**Fig. 2.** Interactive visualization using Momba's simulation engine.

## 4 Harvesting the Benefits

Having constructed the models and gained confidence in their adequacy, we are now ready to harvest the benefits of formal modeling and to apply various state-of-the-art analysis tools, exploiting the JANI-model interchange. Again, Momba provides the necessary functions to define properties and hand our models, with the respective properties attached to them, over to common analysis tools.

Imagine that we are interested in the property $P_{max}(\Diamond \texttt{on\_goal} \wedge \mathit{fuel} > 0)$, i. e., the maximal probability of reaching a goal cell with a non-empty tank from a given start cell. Using Momba's syntax-aware macros, we first construct a disjunction over all goal cells and then define the property using the concise syntax provided by Momba's `prop` function:

```
on_goal = reduce(lor, (expr("car_pos == $g", g=g) for g in goal_cells), False)
define_property(
    prop("min({ Pmax(F($on_goal and fuel > 0)) | initial })", on_goal=on_goal),
    name="goalProbabilityFuel",
)
```

After generating a model with the vehicle starting from position $(0, 7)$ on the track depicted in Fig. 1 and with sand as underground, the value iteration engine mcsta [18] of The Modest Toolset calculates a probability of 87.5 % taking 153 s when invoked by Momba with the model. Momba also cross-checks the results for us, by invoking Storm's dd engine [9] (the fastest engine for this model) and obtains the same result in 107 s. These experiments have been carried out on a standard laptop with an Intel Core i7 at 2.7 GHz.

## 5 Conclusion

We presented Momba, a Python framework for dealing with quantitative models covering the whole process of model creation, validation, and analysis providing an integrated and intuitive experience. In a user story on Racetrack, we demonstrated how Momba's capabilities can be used throughout all stages of the development process of cyber-physical models.

We demonstrated how Momba enables scenario-based model construction with Python code in a concise and modular way with syntax-aware macros. Using Momba's simulation engine, we were able to rapidly prototype an interactive visualization thereby gaining confidence in our models and, finally, thanks to JANI-model, we demonstrated how to analyse our models with state-of-the-art model checkers directly invoked and cross-checked by Momba.

By basing Momba on Python, we aim to harvest the tools developed by the data-science community. Especially, when combined with Jupyter [26], Momba enables literate programming [32] combining code, data, and documentation for reproducible experiments and process documentation.

We hope that Momba helps to open up the world of formal modeling towards a broader community by lowering or removing barriers otherwise obstructing the application of formal models. Momba's infrastructure is implemented in such a way that it can easily be extended into other directions and for connections to other research areas, e. g., model checking policies machine learned with Python libraries [16,17].
